# Relationships of SGLT2 inhibition, circulating metabolites, and cancer: A Mendelian randomization study

**DOI:** 10.1097/MD.0000000000048480

**Published:** 2026-04-24

**Authors:** Wenhui Li, Hui Zhou, Xiaolei Wang, Wangyang Li, Wenying Xie, Jing Zhang, Linjing Qiu, Changjiang Wu, Taojing Zhang

**Affiliations:** a The Second Clinical Medical School, Beijing University of Chinese Medicine, Beijing, China; b Department of Endocrinology, Dongfang Hospital, Beijing University of Chinese Medicine, Beijing, China.

**Keywords:** cancer, circulating metabolites, Mendelian randomization, SGLT2 inhibition

## Abstract

The effect of sodium-glucose cotransporter 2 (SGLT2) inhibition on cancer remains controversial. This study aimed to investigate the causal relationship between SGLT2 inhibition, circulating metabolites and cancer through Mendelian randomization. Genetic instruments for SGLT2 inhibition were identified as genetic variants. A two-sample, two-step Mendelian randomization approach was adopted to determine the causal relationship between SGLT2 inhibition and cancer, as well as the mediating role of circulating metabolites that link SGLT2 inhibition to cancer. SGLT2 inhibition was associated with an increased risk of lung cancer, colorectal cancer, skin cancer, and basal cell carcinoma, and a decreased risk of brain cancer. The mediation proportions of SGLT2 inhibition through polyunsaturated fatty acids and omega-6 fatty acids concerning colorectal cancer were 2.388% and 2.131% of the total effect, respectively. The mediation proportion of SGLT2 inhibition through acetone concerning skin cancer was 5.127%. For brain cancer, the mediation proportions of SGLT2 inhibition through medium-high-density lipoprotein triglycerides, high-density lipoprotein triglycerides, and very-low-density lipoprotein triglycerides were 2.648%, 2.131%, and 2.095%, respectively. Our study established the causal effects of SGLT2 inhibition on circulating metabolites. SGLT2 inhibition can influence cancer through various metabolites.

## 1. Introduction

Cancer represents a substantial public health challenge, with global statistics indicating that nearly 20 million new cases were diagnosed and 9.7 million deaths attributed to cancer in 2022.^[1]^ Lung cancer is the leading cause of both incidence and mortality, followed closely by female breast cancer, colorectal cancer, prostate cancer, and gastric cancer, all of which show high diagnosis rates. The sodium-glucose cotransporter 2 (SGLT2) is expressed in various cancer cell types, including those found in pancreatic cancer, prostate cancer, and glioblastoma.^[2]^ A study has confirmed that glioma cells utilize SGLT2 for glucose uptake, a process that can be inhibited by the SGLT2 inhibitor canagliflozin.^[3]^

SGLT2 inhibitors constitute a novel class of antidiabetic medications that promote urinary glucose excretion and lower serum glucose levels by inhibiting glucose reabsorption in the proximal renal tubules.^[4]^ Beyond glycemic effects, the role in cardio-renal protection has been well-established, and the potential antitumor effects are increasingly gaining attention. Several studies suggest that SGLT2 inhibitors may reduce the risk of specific cancers, such as cervical, prostate and endometrial cancer;^[5–7]^ however, other studies have indicated a potential association with an increased risk of certain cancers.^[8]^ Current evidence primarily stems from observational studies, which are susceptible to confounding factors (e.g. diabetes duration, glycemic control, and concomitant medications) and reverse causality, making it difficult to establish a causal relationship.

Mendelian randomization (MR) utilizes genetic variants strongly associated with the exposure of interest as instrumental variables. This approach can effectively minimize confounding and reverse causality bias, approximating a randomized controlled trial, and is particularly suitable for assessing the causal relationship between long-term drug effects and outcomes like cancer.

Advances in metabolomics provide a new perspective for uncovering disease mechanisms.^[9]^ An observational study has indicated that metabolic dysregulation is a defining characteristic of cancer, with numerous circulating metabolites implicated in cancer development.^[10]^ Meanwhile, SGLT2 inhibition significantly influences metabolites related to lipids, amino acids, and ketone bodies, suggesting that these metabolites may mediate their association with cancer.^[11–13]^

Based on this, we hypothesized that SGLT2 inhibition might influence cancer risk by regulating circulating metabolites. This study employed a two-sample, two-part MR study to systematically investigate the mediating role of metabolites in the relationship between SGLT2 inhibition and various cancers. It aimed to elucidate the complex interplay between SGLT2 inhibition, circulating metabolites, and cancer risk from a genetic perspective, thereby helping to clarify existing uncertainties in this field.

## 2. Methods

### 2.1. Study design

**Figure 1.**
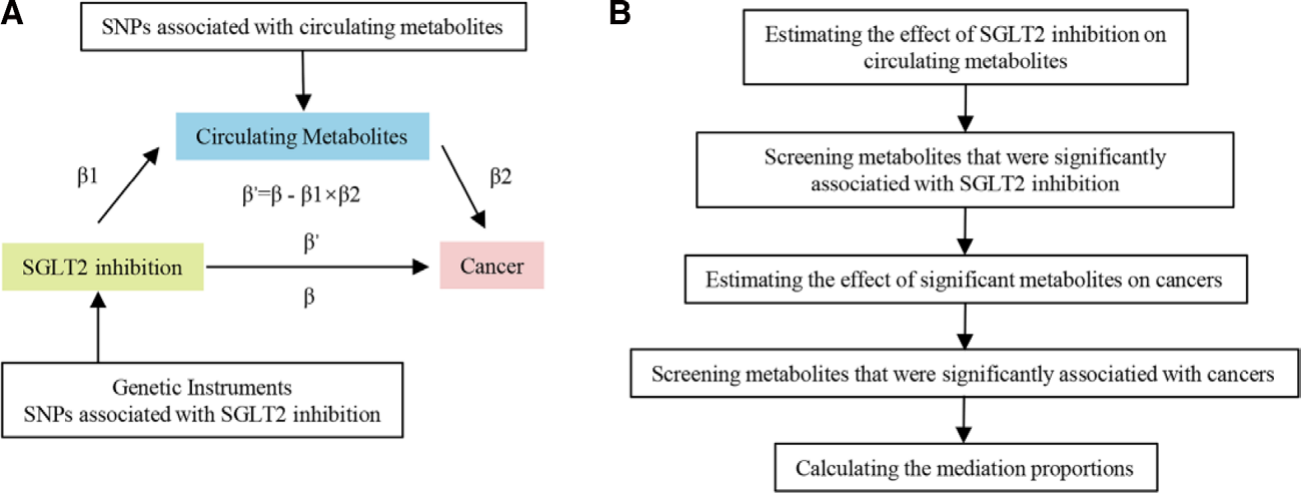
Study design and selection of circulating metabolites. (A) Conceptual framework of the two-step Mendelian randomization (MR) analysis used to evaluate the causal pathways from sodium-glucose cotransporter 2 (SGLT2) inhibition to cancer risk via circulating metabolites. (B) Flow chart outlining the selection process for circulating metabolites included in the two-step MR analysis.

A two-sample MR study was employed to assess the causal relationship between SGLT2 inhibition and cancer. To further explore potential mediating pathways, a two-step MR design was utilized for mediation analysis (Fig. 1A), examining whether circulating metabolites mediate the causal effect of SGLT2 inhibition on cancer. Specifically, we first screened for metabolites that were significantly affected by SGLT2 inhibition, then estimated the effects of these metabolites on cancer, and calculated the mediation proportions (the analysis flow chart is shown in Fig. 1B).

The instrumental variables used in MR must meet 3 key assumptions: the genetic variant is strongly associated with the exposure; the genetic variant is independent of confounders that may affect the relationship between exposure and outcome; and the genetic variant influences the outcome solely through the exposure and not through any alternative causal pathways.

### 2.2. Genetic instruments for SGLT2 inhibition

We selected genetic instruments for SGLT2 inhibition following a previously reported multi-step procedure.^[14]^ Firstly, genetic variants associated with SLC5A2 mRNA expression were selected using data from the Genotype-Tissue Expression Project and eQTLGen Consortium. Secondly, the association between SLC5A2 variants and glycated hemoglobin (HbA1c) levels was estimated utilizing genome-wide association study (GWAS) data from 344,182 nondiabetic individuals of European ancestry from the UK Biobank. This approach helped reduce potential confounding of HbA1c genetic effects by glucose-lowering medications, and single-nucleotide polymorphisms (SNPs) with *P* < 1 × 10^−4^ for association with HbA1c were selected for the next step. Thirdly, Bayesian colocalization analysis was performed to assess whether SLC5A2 expression and HbA1c share a common causal variant. Only SNPs with a posterior colocalization probability exceeding 0.70 were retained. Finally, to satisfy the independence assumption of instrumental variables, a standard clumping procedure was applied using the 1000 Genomes Project European sample as a reference panel. Clumping was performed with a linkage disequilibrium threshold of *r*^2^ < 0.8, retaining the most representative SNP from each correlated set. To validate the selected genetic instruments for SGLT2 inhibition, we performed a positive control analysis using type 2 diabetes mellitus (T2DM) GWAS data from the Integrative Epidemiology Unit (IEU) Open GWAS database (Table 1).

**Table 1:** Summary of GWAS data sources and sample characteristics.

	GWAS ID	PMID	Sample size	Ancestry
SGLT2 inhibition	NA	37848934	344,182	European
T2DM	ebi-a-GCST90018926	34594039	490,089	European
Lung cancer	ebi-a-GCST90018875	34594039	492,803	European
Hepatic cancer	ebi-a-GCST90018858	34594039	475,638	European
Hepatic bile duct cancer	ebi-a-GCST90018803	34594039	476,091	European
Esophageal cancer	ebi-a-GCST90018841	34594039	476,306	European
Gastric cancer	ebi-a-GCST90018849	34594039	476,116	European
Pancreatic cancer	ebi-a-GCST90018893	34594039	476,245	European
Colorectal cancer	ebi-a-GCST90018808	34594039	470,002	European
Thyroid cancer	ebi-a-GCST90018929	34594039	491,974	European
Skin cancer	ebi-a-GCST90018921	34594039	492,203	European
Brain tumor	ebi-a-GCST90018800	34594039	491,542	European
Malignant lymphoma	ebi-a-GCST90018878	34594039	490,803	European
Basal cell carcinoma	ebi-a-GCST90013410	34594039	392,871	European

This table presents the genome-wide association study (GWAS) datasets for SGLT2 inhibition, type 2 diabetes mellitus (T2DM), and 12 cancers, including GWAS ID, PMID, sample size, and ancestry. NA, not available in the summary data.

GWAS = genome-wide association study, SGLT2 = sodium-glucose cotransporter 2, T2DM = type 2 diabetes mellitus.

### 2.3. Genetic instruments for circulating metabolites and cancer

A systematic search was conducted to obtain absolute concentrations and ratios of 249 nuclear magnetic resonance circulating metabolites, lipids, and lipoprotein subcomponents from 121,000 participants in the UK Biobank. The nuclear magnetic resonance metabolomic biomarker data comprised absolute concentrations for 168 biomarkers and ratios for 81 biomarkers, primarily consisting of lipids and lipoprotein subcomponents, as well as fatty acids, amino acids, ketones, and glycolysis-related metabolites.^[15]^ The complete GWAS summary data for these 168 metabolites are publicly accessible through the IEU Open GWAS project database. We restricted the analyses to genetic variants of each biomarker that were at a genome-wide significant (*P* < 5 × 10^−8^) and were independent of each other (linkage disequilibrium, *r*^2^ < 0.8 within 250 kb). GWAS summary data for the 12 cancers investigated were also sourced from the IEU Open GWAS database, with all data restricted to individuals of European ancestry to ensure a consistent genetic background (detailed sample sizes are provided in Table 1).

### 2.4. Statistical analysis

#### 2.4.1. MR analysis

A two-sample MR analysis was performed to explore the causal relationship between SGLT2 inhibition and 12 common cancers, as well as T2DM. The exposure, metabolite, and outcome data in this design were obtained from independent samples, minimizing bias due to sample overlap. Prior to analysis, stringent allele harmonization was performed to ensure consistent effect allele alignment between exposure and outcome datasets, and SNPs with ambiguous strand orientation were inferred or excluded. The inverse-variance weighted (IVW) method served as the primary analytical approach, as it provides the most efficient estimate when all instrumental variable assumptions are met. To ensure the reliability of the results, the weighted median and MR-Egger methods were also employed.

#### 2.4.2. Mediation analysis

A two-step MR design was utilized for mediation analysis to investigate whether specific circulating metabolites could mediate the causal pathway from SGLT2 inhibition to cancer. Firstly, the effects of SGLT2 inhibition on 168 circulating metabolites were estimated using two-sample MR (denoted as β1 in Fig. 1A). Next, the impacts of those metabolites that demonstrated statistically significant associations with SGLT2 inhibition on cancer were assessed through two-sample MR (denoted as β2 in Fig. 1A). The total effect (denoted as β in Fig. 1A) obtained from the primary MR analyses was decomposed into an indirect effect (calculated as indirect effect = β1×β2) and a direct effect (computed as direct effect = total effect ‐ indirect effect). The proportion mediated was determined by dividing the indirect effect by the total effect, with 95% confidence intervals (CIs) calculated using the delta method.

#### 2.4.3. Sensitivity analysis and quality control

To ensure robustness, we performed comprehensive sensitivity analyses. The strength of the instrumental variables was assessed using the *F*-statistic, and those with an *F*-statistic of <10 were removed. Heterogeneity was evaluated via Cochran *Q* test; if the *P*-value was <.05, a random-effects IVW model was applied. Horizontal pleiotropy was examined using the MR-Egger intercept test, and if the *P*-value was <.05, the Mendelian Randomization Pleiotropy RESidual Sum and Outlier method was used to identify and remove outlier SNPs. Finally, a leave-one-out analysis was performed to determine whether causal estimates were driven by any single influential SNP.

Taken together, these procedures established a two-sample, two-step Mendelian randomization framework that first evaluated the causal effects of genetically proxied SGLT2 inhibition on cancer and circulating metabolites, and then quantified the extent to which selected metabolites mediated these effects. In the following Section 3, we present the primary Mendelian randomization estimates, mediation analyses, and sensitivity analyses derived from this framework.

## 3. Results

### 3.1. Effects of SGLT2 inhibition on cancer and T2DM

Using the two-sample MR framework described, we first assessed the genetic instruments and their association with outcomes. Consistent with our predefined selection criteria, 10 independent SNPs within SLC5A2 were retained as genetic instruments for SGLT2 inhibition, each exhibiting an *F*-statistic >13 (Table 2). In the MR analysis, genetically proxied SGLT2 inhibition was associated with a reduced risk of T2DM (odds ratio [OR] = 0.453, 95% CI 0.321–0.640, *P* < .05).

**Table 2:** Genetic instruments for SGLT2 inhibition derived from GWAS data.

Exposure	SNP	EA	OA	Beta	SE	*P* value	*R* ^2^	*F*-statistic
SGLT2 inhibition	rs111510548	C	T	0.015	0.004	6.69E‐05	0.000	14.06
SGLT2 inhibition	rs8057207	T	C	0.013	0.002	4.55E‐08	0.000	42.25
SGLT2 inhibition	rs9926717	G	A	0.011	0.003	9.61E‐06	0.000	13.44
SGLT2 inhibition	rs116943658	A	G	0.013	0.002	3.01E‐07	0.000	42.25
SGLT2 inhibition	rs13334492	A	G	0.011	0.002	7.57E‐07	0.000	30.25
SGLT2 inhibition	rs2070896	C	T	0.017	0.002	1.70E‐12	0.000	72.25
SGLT2 inhibition	rs28641848	T	C	0.011	0.003	1.21E‐05	0.000	13.44
SGLT2 inhibition	rs28692853	A	C	0.015	0.002	2.78E‐10	0.000	56.25
SGLT2 inhibition	rs67464975	T	C	0.012	0.002	4.03E‐07	0.000	36.00
SGLT2 inhibition	rs8050328	G	T	0.016	0.002	1.09E‐11	0.000	64.00

This table lists the single nucleotide polymorphisms (SNPs) that serve as instrumental variables for SGLT2 inhibition, including effect sizes, standard errors, and allele information.

EA = effect allele, GWAS = genome-wide association study, OA = other allele, SE = standard error, SNPs = single nucleotide polymorphisms.

**Figure 2.**
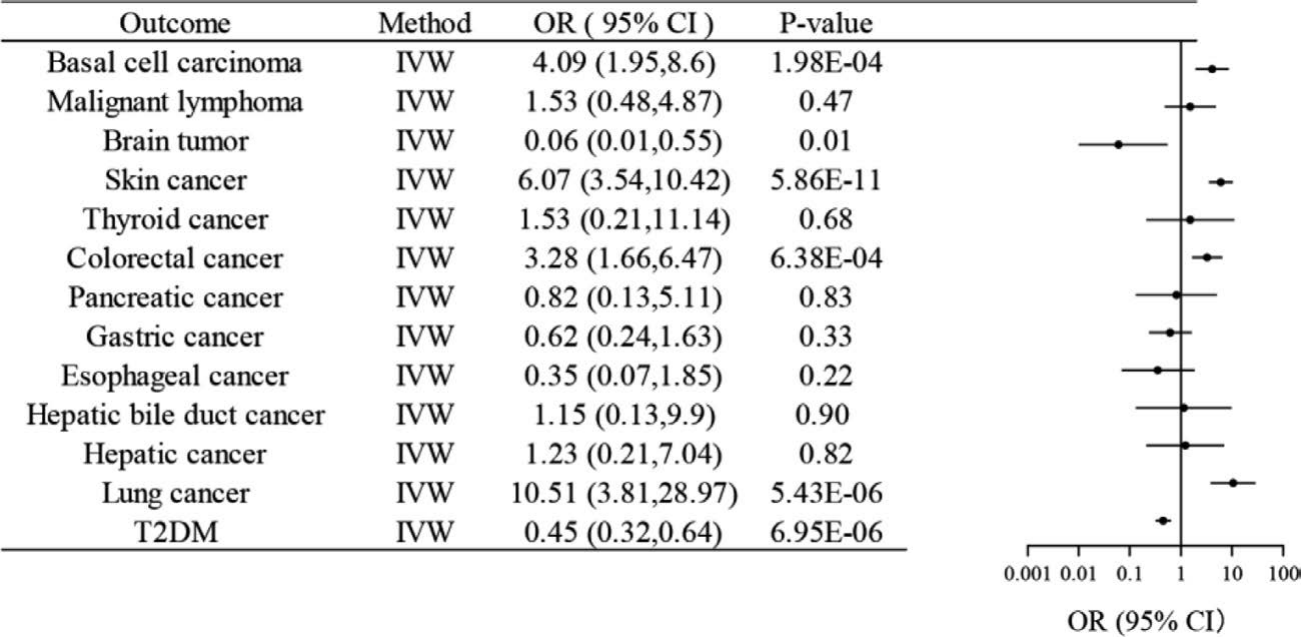
Association of SGLT2 inhibition with 12 cancer and type 2 diabetes. Inverse-variance weighted (IVW) Mendelian randomization estimates are shown as odds ratios (ORs) with 95% confidence intervals (CIs) for the effect of SGLT2 inhibition on the risk of 12 cancers and T2DM. SGLT2 = sodium-glucose cotransporter 2, T2DM = type 2 diabetes mellitus.

Figure 2 summarizes the primary Mendelian randomization study results for SGLT2 inhibition and multiple cancer risks. Genetically predicted SGLT2 inhibition was significantly associated with an increased risk of 4 cancers (lung, colorectal, skin, and basal cell carcinoma) and a decreased risk of brain cancer. As follows: lung cancer (OR = 10.51; 95% CI, 3.81–28.97, *P *< .05), colorectal cancer (OR = 3.28; 95% CI, 1.66–6.47, *P* < .05), skin cancer (OR = 6.07; 95% CI, 3.54–10.42, *P* < .05), basal cell carcinoma (OR = 4.09; 95% CI, 1.95–8.60, *P* < .05), and brain cancer (OR = 0.06; 95% CI, 0.01–0.55, *P* < .05).

**Figure 3.**
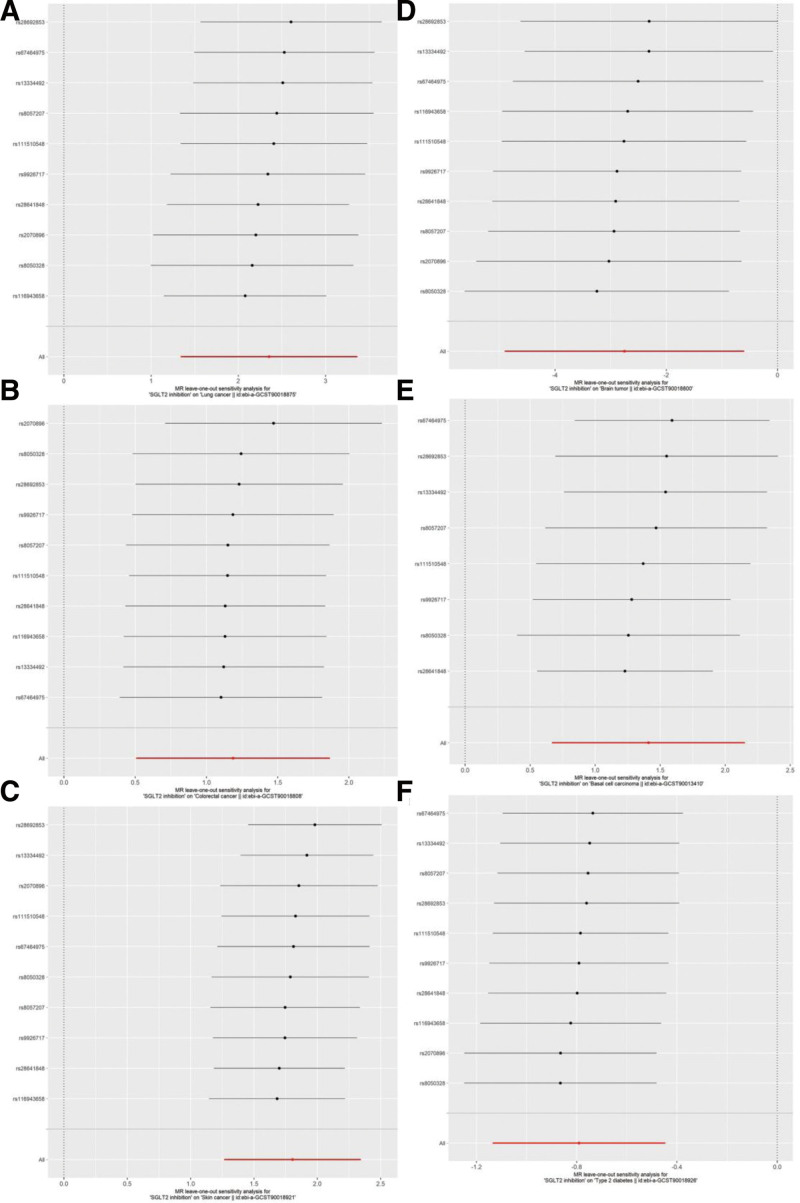
Leave-one-out analysis of SGLT2 inhibition for selected cancers and type 2 diabetes. Leave-one-out analyses evaluate the influence of individual SNPs on the association between SGLT2 inhibition and (A) lung cancer, (B) colorectal cancer, (C) skin cancer, (D) brain tumor, (E) basal cell carcinoma, and (F) type 2 diabetes mellitus. SGLT2 = sodium-glucose cotransporter 2, SNPs = single nucleotide polymorphisms.

Sensitivity analyses supported the robustness of these findings. Cochran *Q* test for heterogeneity did not indicate any significant between-SNP heterogeneity, and the MR-Egger intercept test showed no evidence of horizontal pleiotropy (all *P* > .05; Table 3). Leave-one-out analysis confirmed that no single SNP drove the overall associations (Fig. 3).

**Table 3:** Sensitivity analyses for the association between SGLT2 inhibition and 12 cancers.

Exposure	SNPs (n)	Cochran *Q* test		MR-Egger	
		*Q*	*P*-value	Egger intercept	*P*-value
Lung cancer	9	11.65	0.23	‐2.06E‐02	.66
Hepatic cancer	9	8.90	0.45	‐1.68E‐01	.05
Hepatic bile duct cancer	9	3.92	0.92	‐1.75E‐02	.86
Esophageal cancer	9	3.50	0.94	‐8.68E‐02	.26
Gastric cancer	9	3.00	0.96	9.70E‐03	.82
Pancreatic cancer	9	4.15	0.90	-4.74E‐02	.57
Colorectal cancer	9	4.59	0.87	4.89E‐02	.13
Thyroid cancer	9	5.34	0.80	‐1.33E‐02	.88
Skin cancer	9	11.68	0.23	2.19E‐02	.39
Brain tumor	9	5.16	0.82	‐6.88E‐02	.48
Malignant lymphoma	9	0.74	1.00	‐1.50E‐02	.78
Basal cell carcinoma	7	9.83	0.20	0.00	.95

This table reports heterogeneity and pleiotropy assessments for the MR estimates linking SGLT2 inhibition to each cancer, including Cochran *Q* statistics and MR-Egger intercept tests. Cochran *Q*: assesses heterogeneity among SNPs. *P* > .05 indicates no significant heterogeneity. Egger intercept: tests for pleiotropy. *P* > .05 indicates no significant pleiotropic bias.

MR = Mendelian randomization, SGLT2 = sodium-glucose cotransporter 2, SNPs = single-nucleotide polymorphisms.

### 3.2. Effects of SGLT2 inhibition on circulating metabolites

To investigate the metabolic mechanisms through which SGLT2 inhibition influences cancer risk, we systematically estimated the effects of SGLT2 inhibition on 168 circulating metabolites and identified significant associations with 53 metabolites (*P* < .01). These metabolites primarily included amino acids, cholesterol, phosphatidylcholines, compounds, esterified cholesterol, fatty acids, free cholesterol, glycolysis, ketones, lipoprotein particles, phospholipids, size & apo-lipoprotein, total lipids, and triglycerides (TGs). Of these, 40 metabolites were positively correlated with SGLT2 inhibition, while 13 showed negative correlations (Table 4).

**Table 4:** Effects of SGLT2 inhibition on circulating metabolites in the first step of the two-step MR.

Circulating metabolites	OR (95% CI)	*P*
**Amino acids**		
Alanine	1.40 (1.14–1.72)	1.17E‐03
Glycine	1.41 (1.16–1.72)	4.76E‐04
Histidine	0.73 (0.59–0.89)	2.46E‐03
Isoleucine	0.71 (0.58–0.87)	8.37E‐04
**Cholesterol**		
Cholesterol in medium HDL	1.35 (1.12–1.63)	1.97E‐03
Cholesterol in small HDL	1.91 (1.56–2.35)	5.89E‐10
Cholesterol in very large HDL	0.59 (0.47–0.75)	1.52E‐05
**Cholines**		
Phosphatidylcholines	1.44 (1.19–1.75)	1.83E‐04
Phosphoglycerides	1.41 (1.16–1.71)	5.33E‐04
Total cholines	1.38 (1.14–1.67)	1.11E‐03
**Compounds**		
Total concentration of lipoprotein particles	1.52 (1.25–1.85)	2.10E‐05
Total triglycerides	1.33 (1.09–1.63)	4.61E‐03
**Esterified cholesterol**		
Cholesteryl esters in large LDL	1.33 (1.08–1.63)	6.56E‐03
Cholesteryl esters in medium HDL	1.36 (1.12–1.64)	1.51E‐03
Cholesteryl esters in small HDL	1.90 (1.54–2.33)	1.14E‐09
Cholesteryl esters in very large HDL	0.62 (0.5–0.78)	5.52E‐05
**Fatty acids**		
Total fatty acids	1.40 (1.14–1.71)	1.36E‐03
Degree of unsaturation	0.76 (0.63–0.93)	6.39E‐03
Linoleic acid	1.39 (1.14–1.70)	1.36E‐03
Monounsaturated fatty acids	1.49 (1.21–1.82)	1.29E‐04
Omega-6 fatty acids	1.38 (1.13–1.69)	1.58E‐03
Polyunsaturated fatty acids	1.32 (1.08–1.61)	7.17E‐03
Saturated fatty acids	1.32 (1.08–1.62)	7.53E‐03
**Free cholesterol**		
Free cholesterol in medium HDL	1.29 (1.07–1.55)	7.83E‐03
Free cholesterol in small HDL	1.78 (1.45–2.18)	2.28E‐08
Free cholesterol in very large HDL	0.52 (0.41–0.67)	5.22E‐07
**Glycolysis**		
Citrate	0.72 (0.58–0.88)	1.38E‐03
**Ketone bodies**		
Acetone	0.64 (0.51–0.80)	6.77E‐05
Glycoprotein acetyls	1.34 (1.09–1.64)	5.24E‐03
Pyruvate	0.61 (0.50–0.75)	2.99E‐06
**Lipoprotein particles**		
Concentration of HDL particles	1.54 (1.27–1.86)	1.28E‐05
Concentration of medium HDL particles	1.37 (1.13–1.65)	1.18E‐03
Concentration of small HDL particles	1.90 (1.55–2.33)	9.28E‐10
Concentration of very large HDL particles	0.61 (0.48–0.78)	7.55E‐05
**Phospholipids**		
Phospholipids in medium HDL	1.44 (1.18–1.74)	2.24E‐04
Phospholipids in medium LDL	1.32 (1.08–1.62)	8.07E‐03
Phospholipids in small HDL	1.84 (1.50–2.26)	4.26E‐09
Phospholipids in very large HDL	0.58 (0.46–0.73)	2.18E‐06
**Size&apo-LP**		
Average diameter for HDL particles	0.72 (0.59–0.88)	1.51E‐03
Average diameter for LDL particles	1.36 (1.11–1.66)	2.85E‐03
Average diameter for VLDL particles	1.32 (1.09–1.60)	4.58E‐03
Apolipoprotein A1	1.32 (1.10–1.6)	3.35E‐03
**Total lipids**		
Total lipids in large VLDL	1.31 (1.07–1.60)	7.53E‐03
Total lipids in medium HDL	1.42 (1.17–1.72)	3.15E‐04
Total lipids in small HDL	1.92 (1.56–2.35)	5.01E‐10
Total lipids in very large HDL	0.59 (0.46–0.74)	7.32E‐06
**Triglycerides**		
Triglycerides in HDL	1.31 (1.07–1.61)	8.42E‐03
Triglycerides in large VLDL	1.37 (1.12–1.67)	1.86E‐03
Triglycerides in medium HDL	1.37 (1.12–1.68)	2.51E‐03
Triglycerides in medium VLDL	1.39 (1.14–1.70)	1.15E‐03
Triglycerides in small HDL	1.46 (1.20–1.79)	1.82E‐04
Triglycerides in small VLDL	1.37 (1.12–1.67)	2.04E‐03
Triglycerides in VLDL	1.35 (1.11–1.64)	3.07E‐03

This table summarizes the causal estimates for the association of SGLT2 inhibition with circulating metabolites, expressed as odds ratios (ORs) with 95% confidence intervals (CIs).

CI = confidence interval, HDL = high-density lipoprotein, LDL = low-density lipoprotein, MR = Mendelian randomization, OR = odds ratio, SGLT2 = sodium-glucose cotransporter 2, size&apo-LP = size & apo-lipoprotein, VLDL = very-low-density lipoprotein.

### 3.3. Metabolites associated with cancer risks

We then examined the associations between these 53 metabolites and the 5 cancers. We found that 24 metabolites were significantly associated with at least one cancer (*P* < .01). These metabolites were further categorized as follows: 21 metabolites demonstrated causal relationships with specific cancer: 4 for lung cancer, 14 for colorectal cancer, 1 for skin cancer, and 2 for brain cancer. Three metabolites associated with multiple cancer: degree of unsaturation was positively correlated with lung cancer (OR = 1.12; 95% CI, 1.07–1.19, *P* < .05), colorectal cancer (OR = 1.11; 95% CI, 1.06–1.16, *P* < .05), and skin cancer (OR = 1.12; 95% CI, 1.09–1.16, *P* < .05). Glycoprotein acetyls were inversely associated with lung (OR = 0.89; 95% CI, 0.82–0.97, *P* < .05) and skin cancer (OR = 0.92; 95% CI, 0.87–0.97, *P* < .05]. Very-low-density lipoprotein triglycerides (VLDL-TG) showed protective associations with lung (OR = 0.92; 95% CI, 0.87–0.98, *P* < .05) and brain cancer (OR = 0.82; 95% CI, 0.71–0.95, *P* < .05). All association results are detailed in Table 5.

**Table 5:** Associations between circulating metabolites and cancer outcomes in the second step of the two-step MR.

Metabolites	OR (95% CI)	*P*-value
**Lung cancer**		
Degree of unsaturation	1.12 (1.07–1.19)	1.32E‐05
Linoleic acid	0.88 (0.81–0.96)	2.67E‐03
Monounsaturated fatty acids	0.90 (0.84–0.97)	4.86E‐03
Glycoprotein acetyls	0.89 (0.82–0.97)	6.74E‐03
Average diameter for LDL particles	0.87 (0.76–0.96)	9.61E‐03
Triglycerides in VLDL	0.92 (0.87–0.98)	7.33E‐03
Triglycerides in large VLDL	0.92 (0.86–0.97)	4.67E‐03
**Colorectal cancer**		
Cholesterol in small HDL	0.89 (0.82–0.96)	1.92E‐03
Cholesterol in very large HDL	1.11 (1.07–1.16)	3.90E‐07
Cholesteryl esters in small HDL	0.90 (0.84–0.96)	1.89E‐03
Cholesteryl esters in very large HDL	1.11 (1.06–1.15)	1.57E‐06
Degree of unsaturation	1.11 (1.06–1.16)	1.71E‐05
Omega-6 fatty acids	1.08 (1.02–1.15)	8.48E‐03
Polyunsaturated fatty acids	1.11 (1.05–1.17)	2.84E‐04
Free cholesterol in very large HDL	1.10 (1.06–1.14)	3.44E‐06
Concentration of small HDL particles	0.88 (0.82–0.95)	9.97E‐04
Concentration of very large HDL particles	1.11 (1.06–1.15)	1.15E‐06
Phospholipids in small HDL	0.91 (0.85–0.97)	2.90E‐03
Phospholipids in very large HDL	1.11 (1.07–1.16)	4.19E‐07
Average diameter for HDL particles	1.08 (1.04–1.13)	7.47E‐05
Total lipids in small HDL	0.89 (0.83–0.95)	8.36E‐04
Total lipids in very large HDL	1.11 (1.07–1.16)	3.53E‐07
**Skin cancer**		
Degree of unsaturation	1.12 (1.09–1.16)	3.75E‐12
Acetone	0.81 (0.70–0.95)	9.03E‐03
Glycoprotein acetyls	0.92 (0.87–0.97)	2.26E‐03
**Brain tumor**		
Triglycerides in HDL	0.81 (0.71–0.91)	8.27E‐04
Triglycerides in VLDL	0.82 (0.71–0.95)	9.17E‐03
Triglycerides in medium HDL	0.79 (0.70–0.90)	3.65E‐04

This table presents MR estimates for the associations of selected circulating metabolites with lung, colorectal, skin, and brain cancers. Degree of unsaturation was positively associated with lung, colorectal, and skin cancer; glycoprotein acetyls were negatively associated with lung and skin cancer; and triglycerides in very-low-density lipoprotein (VLDL) were inversely associated with lung and brain cancer.

95% CIs = 95% confidence intervals, HDL = high-density lipoprotein, LDL = low-density lipoprotein, MR = Mendelian randomization, OR = odds ratio, VLDL = very-low-density lipoprotein.

### 3.4. Mediation analysis results

**Figure 4.**
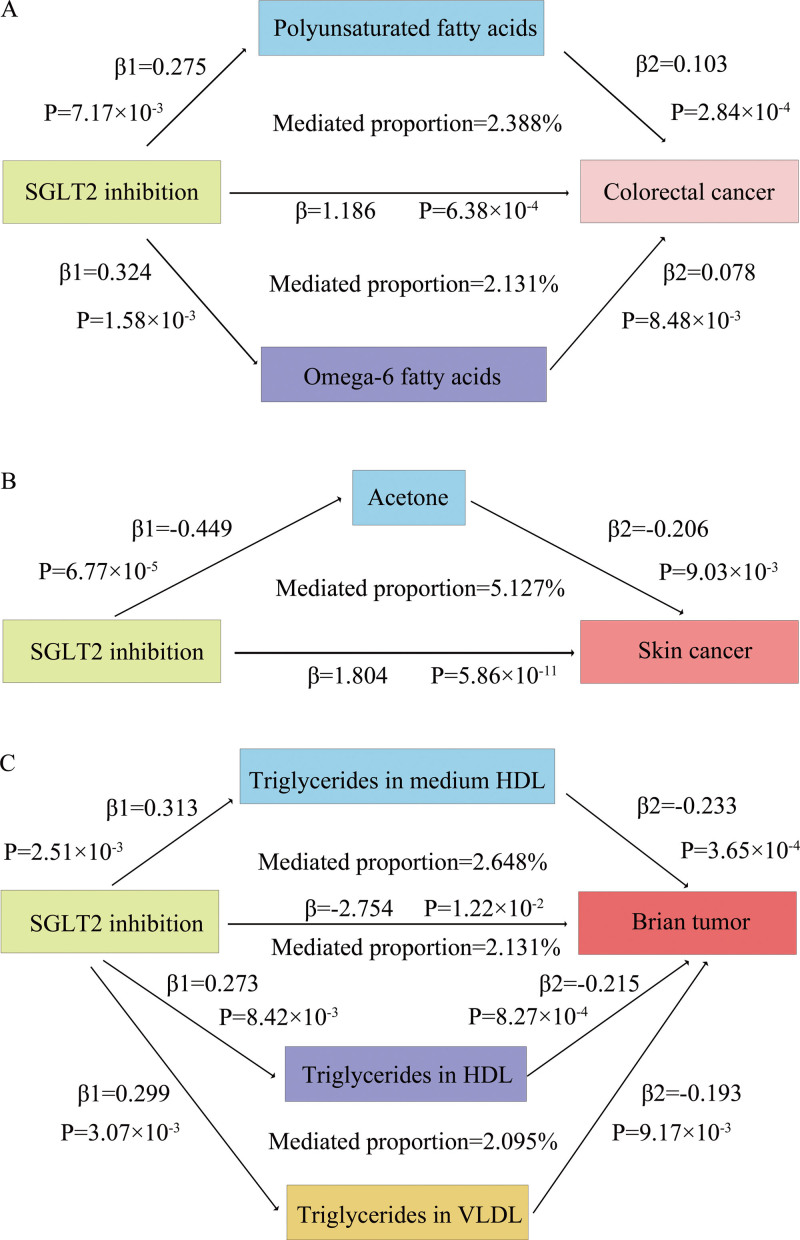
Mediating metabolic pathways linking SGLT2 inhibition to cancer risk. Significant metabolite-mediated pathways identified by two-step Mendelian randomization are illustrated for the effects of genetically proxied SGLT2 inhibition on (A) colorectal cancer via polyunsaturated and omega-6 fatty acids, (B) skin cancer via acetone, and (C) brain cancer via triglycerides in M-HDL, HDL, and VLDL. HDL = high-density lipoprotein, M-HDL = medium-high-density lipoprotein, SGLT2 = sodium-glucose cotransporter 2, VLDL = very-low-density lipoprotein.

The principal findings from our mediation analysis were as follows. Statistically significant mediation effects were identified for colorectal cancer, skin cancer, and brain cancer, but not for lung cancer (Table 6 and Fig. 4). In colorectal cancer (total effect β = 1.186), polyunsaturated fatty acids (PUFAs) (β′ = 0.028) and omega-6 fatty acids (β′ = 0.025) mediated 2.388% and 2.131% of the total effect, respectively. In skin cancer (total effect β = 1.804), acetone mediated 5.127% of the total effect. In brain cancer (total effect β = −2.754), medium-high-density lipoprotein (M-HDL) triglycerides (β′ = −0.073), high-density lipoprotein triglycerides (β′ = −0.059), and VLDL-TG (β′ = −0.058) mediated 2.648%, 2.131%, and 2.095%, respectively. In contrast, for lung cancer (total effect β = 2.352), all candidate metabolites had effects opposite in direction to the total effect (e.g., degree of unsaturation β′ = −0.032) and therefore did not meet the criteria for valid mediation.

**Table 6:** Mediation analysis of circulating metabolites in the pathway from SGLT2 inhibition to cancer.

Exposure	Mediator	Outcome	Total effect		Step 1		Step 2		Mediation	Mediation proportion
			β	*P*-value	β1	*P*-value1	β2	*P*-value2		
SGLT2 inhibition	Degree of unsaturation	Lung cancer	2.352	5.43E‐06	‐0.272	6.39E‐03	0.118	1.32E‐05	‐0.032	‐1.365%
SGLT2 inhibition	Linoleic acid	Lung cancer	2.352	5.43E‐06	0.330	1.36E‐03	‐0.127	2.67E‐03	‐0.042	‐1.782%
SGLT2 inhibition	Triglycerides in large VLDL	Lung cancer	2.352	5.43E‐06	0.314	1.86E‐03	‐0.088	4.67E‐03	‐0.028	‐1.175%
SGLT2 inhibition	Monounsaturated fatty acids	Lung cancer	2.352	5.43E‐06	0.398	1.29E‐04	‐0.101	4.86E‐03	‐0.040	‐1.709%
SGLT2 inhibition	Glycoprotein acetyls	Lung cancer	2.352	5.43E‐06	0.291	5.24E‐03	‐0.114	6.74E‐03	‐0.033	‐1.410%
SGLT2 inhibition	Triglycerides in VLDL	Lung cancer	2.352	5.43E‐06	0.299	3.07E‐03	‐0.082	7.33E‐03	‐0.025	‐1.042%
SGLT2 inhibition	Average diameter for LDL particles	Lung cancer	2.352	5.43E‐06	0.306	2.85E‐03	‐0.154	9.61E‐03	‐0.047	‐2.004%
SGLT2 inhibition	Total lipids in very large HDL	Colorectal cancer	1.186	6.38E‐04	‐0.534	7.32E‐06	0.107	3.53E‐07	‐0.057	‐4.818%
SGLT2 inhibition	Cholesterol in very large HDL	Colorectal cancer	1.186	6.38E‐04	‐0.522	1.52E‐05	0.106	3.90E‐07	‐0.055	‐4.665%
SGLT2 inhibition	Phospholipids in very large HDL	Colorectal cancer	1.186	6.38E‐04	‐0.544	2.18E‐06	0.105	4.19E‐07	‐0.057	‐4.816%
SGLT2 inhibition	Concentration of very large HDL particles	Colorectal cancer	1.186	6.38E‐04	‐0.492	7.55E‐05	0.101	1.15E‐06	‐0.050	‐4.190%
SGLT2 inhibition	Cholesteryl esters in very large HDL	Colorectal cancer	1.186	6.38E‐04	‐0.472	5.52E‐05	0.101	1.57E‐06	‐0.048	‐4.020%
SGLT2 inhibition	Free cholesterol in very large HDL	Colorectal cancer	1.186	6.38E‐04	‐0.648	5.22E‐07	0.094	3.44E‐06	‐0.061	‐5.136%
SGLT2 inhibition	Degree of unsaturation	Colorectal cancer	1.186	6.38E‐04	‐0.272	6.39E‐03	0.101	1.71E‐05	‐0.027	‐2.316%
SGLT2 inhibition	Average diameter for HDL particles	Colorectal cancer	1.186	6.38E‐04	‐0.322	1.51E‐03	0.080	7.47E‐05	‐0.026	‐2.172%
SGLT2 inhibition	Polyunsaturated fatty acids	Colorectal cancer	1.186	6.38E‐04	0.275	7.17E‐03	0.103	2.84E‐04	0.028	2.388%
SGLT2 inhibition	Total lipids in small HDL	Colorectal cancer	1.186	6.38E‐04	0.650	5.01E‐10	‐0.118	8.36E‐04	‐0.077	‐6.467%
SGLT2 inhibition	Concentration of small HDL particles	Colorectal cancer	1.186	6.38E‐04	0.642	9.28E‐10	‐0.126	9.97E‐04	‐0.081	‐6.821%
SGLT2 inhibition	Cholesteryl esters in small HDL	Colorectal cancer	1.186	6.38E‐04	0.640	1.14E‐09	‐0.105	1.89E‐03	‐0.067	‐5.666%
SGLT2 inhibition	Cholesterol in small HDL	Colorectal cancer	1.186	6.38E‐04	0.649	5.89E‐10	‐0.119	1.92E‐03	‐0.077	‐6.512%
SGLT2 inhibition	Phospholipids in small HDL	Colorectal cancer	1.186	6.38E‐04	0.611	4.26E‐09	‐0.099	2.90E‐03	‐0.060	‐5.100%
SGLT2 inhibition	Omega-6 fatty acids	Colorectal cancer	1.186	6.38E‐04	0.324	1.58E‐03	0.078	8.48E‐03	0.025	2.131%
SGLT2 inhibition	Degree of unsaturation	Skin cancer	1.804	5.86E‐11	‐0.272	6.39E‐03	0.115	3.75E‐12	‐0.031	‐1.734%
SGLT2 inhibition	Glycoprotein acetyls	Skin cancer	1.804	5.86E‐11	0.291	5.24E‐03	‐0.085	2.26E‐03	‐0.025	‐1.371%
SGLT2 inhibition	Acetone	Skin cancer	1.804	5.86E‐11	‐0.449	6.77E‐05	‐0.206	9.03E‐03	0.092	5.127%
SGLT2 inhibition	Triglycerides in medium HDL	Brain tumor	-2.754	1.22E‐02	0.313	2.51E‐03	‐0.233	3.65E‐04	‐0.073	2.648%
SGLT2 inhibition	Triglycerides in HDL	Brain tumor	-2.754	1.22E‐02	0.273	8.42E‐03	‐0.215	8.27E‐04	‐0.059	2.131%
SGLT2 inhibition	Triglycerides in VLDL	Brain tumor	-2.754	1.22E‐02	0.299	3.07E‐03	‐0.193	9.17E‐03	‐0.058	2.095%

This table reports the total effect of SGLT2 inhibition on cancer, the effect on circulating metabolites (Step 1), the effect of metabolites on cancer (Step 2), and the estimated mediation effect of metabolites on the SGLT2 inhibition–cancer relationship.

HDL = high-density lipoprotein, LDL = low-density lipoprotein, SGLT2 = sodium-glucose cotransporter 2, VLDL = very-low-density lipoprotein.

The mediation analysis showed that specific circulating metabolites (such as PUFAs, omega-6 fatty acids, acetone, and certain high-density lipoprotein [HDL]/VLDL-TGs) mediated approximately 2% to 5% of the total effect between SGLT2 inhibition and certain cancers. This mediation percentage represents the proportion of the total effect explained through the metabolite-mediated pathway, thereby providing quantitative support for their intermediary role in the causal chain. Therefore, these findings suggested that metabolic pathways may serve as a potential mechanism underlying the observed associations, though the full biological processes require further elucidation.

## 4. Discussion

In summary, this two-sample, two-step MR study demonstrated that genetically proxied SGLT2 inhibition was causally related to the risk of several cancers and that specific circulating metabolites partially mediate these relationships. Specifically, SGLT2 inhibition was associated with an increased risk of lung, colorectal, skin, and basal cell cancers, but a decreased risk of brain cancer. Mediation analysis indicated that PUFAs and omega-6 fatty acids accounted for 2.388% and 2.131% of the increased colorectal cancer risk, respectively; acetone mediated 5.127% of the decreased skin cancer risk; and TGs in M-HDL, HDL, and VLDL accounted for 2.648%, 2.131%, and 2.095% of the protective effect on brain cancer, respectively.

### 4.1. Relationship between SGLT2 inhibition and cancer

The impact of SGLT2 inhibition on cancer has been a subject of controversy. Initially, these inhibitors were associated with an increased incidence of bladder cancer^[16]^; however, subsequent clinical trials and meta-analyses did not support this finding.^[17,18]^ A recent MR study suggested that long-term SGLT2 inhibitors exposure may elevate bladder cancer risk, speculating that its underlying biological effects may vary based on treatment duration and population characteristics.^[19]^ Most studies indicate that SGLT2 inhibitors could lower the risk of lung cancer, especially non-small cell lung cancer (NSCLC). For example, a large-scale Surveillance, Epidemiology and End Results-Medicare linked data study found that SGLT2 inhibitor use was associated with improved overall survival in NSCLC patients with preexisting diabetes, independent of demographic factors, cancer characteristics, and treatment modalities.^[20]^ Another meta-analysis of 29 large-scale trials also demonstrated a significant association between SGLT2 inhibitor use and a reduced incidence of NSCLC.^[21]^ Furthermore, SGLT2 has been identified as an important diagnostic marker in lung premalignancy and early lung adenocarcinoma, with evidence showing that SGLT2 inhibitors can delay the development and growth of lung adenocarcinoma in mouse models.^[22]^ However, a large cohort study found no short-term reduction in lung cancer risk associated with SGLT2 inhibitors compared to dipeptidyl peptidase-4 inhibitors.^[23]^ Our finding of an increased lung cancer risk contrasted with much of the existing literature; this discrepancy may arise from methodological differences, population heterogeneity, or the complex role of SGLT2 across disease stages.

Observational studies found that SGLT2 inhibitors were associated with improved survival and a reduced risk of developing colorectal cancer.^[24,25]^ Experimental evidence indicates that SGLT2 inhibitors may mediate metabolic changes involving SIRT3 upregulation, cell cycle arrest, apoptotic cell death, autophagy, and endoplasmic reticulum stress in colorectal cancer cells.^[26]^ However, our genetic study found an association between SGLT2 inhibition and an increased risk of colorectal cancer. Our genetic analysis, however, found an association with increased colorectal cancer risk. This divergence may reflect confounding in observational studies (e.g., indication bias) versus the focus on long-term biological effects in MR.

A meta-analysis found no significant association between SGLT2 inhibitors and melanoma risk, but found a potential reduced risk of nonmelanoma skin cancer with short-term use.^[27]^ In contrast, our research indicated that SGLT2 inhibition was associated with an increased risk of skin cancer. This finding requires further validation in future studies, which should also consider the potential influence of key confounding factors such as ultraviolet exposure and skin type.

### 4.2. Relationship between SGLT2 inhibition and circulating metabolites

The effects of SGLT2 inhibition on lipid metabolism are indeed controversial. A study found that SGLT2 inhibitor treatment reduced levels of total cholesterol, low-density lipoprotein cholesterol, and TGs,^[28]^ whereas a meta-analysis reported increases in these lipids as well as in high-density lipoprotein cholesterol.^[29]^ Additionally, canagliflozin treatment increased cholesterol content in very-large and large HDL,^[30]^ indicating that the specific characteristics of lipid particles should be considered when investigating its relationship with diseases.

Our study found that SGLT2 inhibition significantly influenced HDL subclass metabolism. Specifically, SGLT2 inhibition increased cholesterol, esterified cholesterol, free cholesterol, lipoprotein particles, phospholipids, and total lipids in small HDL while decreasing these components in very large HDL. Additionally, SGLT2 inhibition elevated fatty acids and TGs, accompanied by a decrease in ketone body-related metabolites. These changes may be partly mediated by mechanisms such as the inhibition of angiopoietin-like protein 3 expression, elevation of plasma campesterol, or lipid mobilization linked to weight loss.^[31–33]^ Animal studies also showed that SGLT2 inhibitors could elevate PUFA metabolites in the myocardium of rats and reduce pyruvate dehydrogenase activity, thereby inhibiting pyruvate utilization.^[34,35]^

### 4.3. Role of circulating metabolites in the relationship between SGLT2 inhibition and cancer

Numerous studies indicate that cancer cells exhibit abnormal lipid metabolism, often synthesizing more fatty acids. In colon cancer development, the metabolism of PUFAs is particularly relevant.^[36]^ Eicosanoids derived from omega-6 and omega-3 fatty acids, specifically those synthesized from arachidonic acid (AA), have pro-inflammatory properties, while those from eicosapentaenoic acid are anti-inflammatory. Imbalances in the AA/eicosapentaenoic acid ratio, especially in metastatic colorectal cancer, present promising biomarkers.

Our genetic finding suggested that SGLT2 inhibition may increase the risk of colorectal cancer by modulating PUFA and omega-6 levels, which is consistent with a previous MR study.^[37]^ That study emphasized omega-6 PUFAs, such as AA, as potential mediators of colorectal cancer, indicating that the PUFA biosynthesis pathway could be a target for preventive interventions. Furthermore, 2 additional studies support an adverse association between specific omega-6 PUFAs and colorectal cancer risk, further reinforcing its biological plausibility.^[38,39]^

Ketone bodies play a dual role in cancer cell metabolism as an alternative energy source. While ketogenic diets have demonstrated antitumor effects in some models, other studies found that a high-fat ketogenic diets promoted melanoma growth in mice by increasing acetoacetate levels.^[40]^ In our study, acetone mediated the partially protective effects of SGLT2 inhibition against skin cancer, though the underlying mechanism remains unclear and may involve the local metabolic microenvironment or specific cell types, requiring further investigation.

Previous research suggests that TGs can increase the risk of various cancers by facilitating lipid metabolic reprogramming.^[41]^ In contrast, we observed that the TG content of M-HDL, HDL, and VLDL subclasses may confer a protective effect of SGLT2 inhibition against brain cancer. Direct studies on the relationship between TGs and brain cancer are currently limited. Whether the protective mechanism is related to the transport of lipids across the blood–brain barrier, brain tissue-specific metabolic pathways, or energy utilization patterns of glial cells requires further exploration in future basic and clinical research.

### 4.4. Strengths and limitations

This study was the first to employ MR analysis to investigate the relationship between SGLT2 inhibition, circulating metabolites, and cancer. Our findings underscored the significant role of lipid metabolism. However, the following limitations should be considered when interpreting the results. First, MR analysis revealed the lifelong effects of SGLT2 inhibition, which might differ from those observed with short-term exposure. Second, the study lacked subgroup analyses by specific SGLT2 inhibitor types or detailed cancer molecular subtypes, preventing the exclusion of potential influences from drug heterogeneity or distinct cancer subtypes. Third, our data were derived exclusively from individuals of European ancestry, which may restrict the generalizability of our findings to broader populations. Finally, although the mediation analysis suggested potential mediating effects for certain metabolites, the effect sizes were small, and the analysis itself relied on multiple assumptions. Therefore, these mediation results should be interpreted cautiously and not as definitive evidence for causal pathways.

We assessed the validity of our instrumental variables and the robustness of the findings using several methods, including IVW, MR-Egger, and Mendelian Randomization Pleiotropy RESidual Sum and Outlier. However, the potential for confounding due to pleiotropy or population stratification cannot be entirely excluded. Therefore, these results should be considered as preliminary genetic evidence for the long-term effects of SGLT2 inhibition. Their clinical relevance and specific biological mechanisms require further elucidation through future multicenter, multi-ancestry studies, and experimental validation.

## 5. Conclusion

This genetic study suggests that SGLT2 inhibition may be associated with an increased risk of lung, colorectal, skin, and basal cell carcinoma, while showing a protective association with brain cancer. Circulating metabolites, such as PUFAs, omega-6 fatty acids, acetone, and TG-rich lipoproteins were identified as potential partial mediators. These findings offer new perspectives on the long-term effects and metabolic mechanisms of SGLT2 inhibitors. Inconsistencies with some clinical studies highlight that real-world associations may be influenced by treatment duration, population characteristics, or confounding. Further prospective studies and refined analyses are needed to evaluate the risk-benefit profile of these drugs.

## Acknowledgments

We thank the participants in all the GWASs used in this study and the investigators who made these GWAS data publicly available.

## Author contributions

**Conceptualization:** Hui Zhou, Xiaolei Wang, Taojing Zhang.

**Data curation:** Wenying Xie, Jing Zhang.

**Formal analysis:** Wenhui Li, Wangyang Li.

**Writing – original draft:** Wenhui Li, Wangyang Li.

**Writing – review & editing:** Linjing Qiu, Changjiang Wu.

## References

[1] Bray F Laversanne M Sung H. Global cancer statistics 2022: GLOBOCAN estimates of incidence and mortality worldwide for 36 cancers in 185 countries. CA A Cancer J Clinicians. 2024;74:229–63. 10.3322/caac.21834 38572751

[2] Wright EM. SGLT2 and cancer. Pflugers Arch. 2020;472:1407–14. 32820343 10.1007/s00424-020-02448-4 PMC7462911

[3] Shoda K Tsuji S Nakamura S. Canagliflozin inhibits glioblastoma growth and proliferation by activating AMPK. Cell Mol Neurobiol. 2023;43:879–92. 35435536 10.1007/s10571-022-01221-8 PMC11415156

[4] Vallon V Verma S. Effects of SGLT2 inhibitors on kidney and cardiovascular function. Annu Rev Physiol. 2021;83:503–28. 33197224 10.1146/annurev-physiol-031620-095920 PMC8017904

[5] Tsui TL Ho YC Ueng KC. The lower incidence of cervical cancer in type 2 diabetes mellitus with sodium-glucose cotransporter 2 inhibitors utilization. J Cancer. 2024;15:6196–203. 39513110 10.7150/jca.101165 PMC11540508

[6] Zheng J Lu J Qi J. The effect of SGLT2 inhibition on prostate cancer: Mendelian randomization and observational analysis using electronic healthcare and cohort data. Cell Rep Med. 2024;5:101688. 39168098 10.1016/j.xcrm.2024.101688 PMC11384955

[7] Yang PJ Wang PH Huang JY. The lower incidence of endometrial cancer after sodium-glucose cotransporter 2 inhibitors administration in type 2 diabetes mellitus population: a nationwide cohort study. Int J Med Sci. 2024;21:1408–13. 38903923 10.7150/ijms.95584 PMC11186417

[8] Xu B Zhou J. Sodium-glucose cotransporter 2 inhibitors and renal cancer in the US FDA adverse event reporting system. Eur J Clin Pharmacol. 2024;80:1959–66. 39285057 10.1007/s00228-024-03759-6

[9] Clish CB. Metabolomics: an emerging but powerful tool for precision medicine. Cold Spring Harb Mol Case Stud. 2015;1:a000588. 27148576 10.1101/mcs.a000588 PMC4850886

[10] Pavlova NN Zhu J Thompson CB. The hallmarks of cancer metabolism: still emerging. Cell Metab. 2022;34:355–77. 35123658 10.1016/j.cmet.2022.01.007 PMC8891094

[11] Kappel BA Lehrke M Schütt K. Effect of empagliflozin on the metabolic signature of patients with type 2 diabetes mellitus and cardiovascular disease. Circulation. 2017;136:969–72. 28874423 10.1161/CIRCULATIONAHA.117.029166

[12] Katano S Yano T Kouzu H. Elevated circulating level of β-aminoisobutyric acid (BAIBA) in heart failure patients with type 2 diabetes receiving sodium-glucose cotransporter 2 inhibitors. Cardiovasc Diabetol. 2022;21:285. 36539818 10.1186/s12933-022-01727-x PMC9768967

[13] Szekeres Z Toth K Szabados E. The effects of SGLT2 inhibitors on lipid metabolism. Metabolites. 2021;11:87. 33535652 10.3390/metabo11020087 PMC7912792

[14] Li J Yu Y Sun Y. SGLT2 inhibition, circulating metabolites, and atrial fibrillation: a Mendelian randomization study. Cardiovasc Diabetol. 2023;22:278. 37848934 10.1186/s12933-023-02019-8 PMC10583416

[15] Ritchie SC Surendran P Karthikeyan S. Quality control and removal of technical variation of NMR metabolic biomarker data in ~120,000 UK Biobank participants. Sci Data. 2023;10:64. 36720882 10.1038/s41597-023-01949-y PMC9887579

[16] Tang H Dai Q Shi W Zhai S Song Y Han J. SGLT2 inhibitors and risk of cancer in type 2 diabetes: a systematic review and meta-analysis of randomised controlled trials. Diabetologia. 2017;60:1862–72. 28725912 10.1007/s00125-017-4370-8

[17] Abrahami D Tesfaye H Yin H. Sodium-glucose cotransporter 2 inhibitors and the short-term risk of bladder cancer: an international multisite cohort study. Diabetes Care. 2022;45:2907–17. 36170656 10.2337/dc22-1174 PMC9998845

[18] Spiazzi BF Naibo RA Wayerbacher LF. Sodium-glucose cotransporter-2 inhibitors and cancer outcomes: a systematic review and meta-analysis of randomized controlled trials. Diabetes Res Clin Pract. 2023;198:110621. 36921905 10.1016/j.diabres.2023.110621

[19] Han Z He Y Li X Li S Ai J. Insights into the impact of sodium-glucose cotransporter 2 inhibition on urinary tract malignancy: a two-sample Mendelian randomization. Diabetes Obes Metab. 2024;26:1986–9. 38356116 10.1111/dom.15490

[20] Luo J Hendryx M Dong Y. Sodium-glucose cotransporter 2 (SGLT2) inhibitors and non-small cell lung cancer survival. Br J Cancer. 2023;128:1541–7. 36765176 10.1038/s41416-023-02177-2 PMC10070339

[21] Xiao Y Yang W Wang M. SGLT2 inhibitors may reduce non-small cell lung cancer and not increase various neoplasms including several skin cancers. Endocrine. 2024;86:1190–1. 38849644 10.1007/s12020-024-03914-0

[22] Zhang L Xue B Yu F Yin Y Jin S. Deciphering the causal relationship between sodium-glucose cotransporter 2 inhibition and cancer risks: a comprehensive mendelian randomization study. J Cancer. 2024;15:3903–12. 38911377 10.7150/jca.96435 PMC11190771

[23] Shapiro SB Yin H Yu OHY Azoulay L. Sodium-glucose cotransporter-2 inhibitors and the risk of lung cancer among patients with type 2 diabetes. Br J Clin Pharmacol. 2024;90:1365–70. 38477518 10.1111/bcp.16039

[24] Chiang CH Chiang CH Hsia YP. The impact of sodium-glucose cotransporter-2 inhibitors on outcome of patients with diabetes mellitus and colorectal cancer. J Gastroenterol Hepatol. 2024;39:902–7. 38296226 10.1111/jgh.16498

[25] Chan RNC Chan RNF Chou OHI Tse G Lee S. Lower risks of incident colorectal cancer in SGLT2i users compared to DPP4i users: a propensity score-matched study with competing risk analysis. Eur J Intern Med. 2023;110:125–7. 36732129 10.1016/j.ejim.2023.01.021

[26] Anastasio C Donisi I Del Vecchio V. SGLT2 inhibitor promotes mitochondrial dysfunction and ER-phagy in colorectal cancer cells. Cell Mol Biol Lett. 2024;29:80. 38811901 10.1186/s11658-024-00599-1 PMC11134909

[27] Tang H Yang K Song Y Han J. Meta-analysis of the association between sodium-glucose co-transporter-2 inhibitors and risk of skin cancer among patients with type 2 diabetes. Diabetes Obes Metab. 2018;20:2919–24. 30039616 10.1111/dom.13474

[28] Calapkulu M Cander S Gul OO Ersoy C. Lipid profile in type 2 diabetic patients with new dapagliflozin treatment; actual clinical experience data of six months retrospective lipid profile from single center. Diabetes Metab Syndr. 2019;13:1031–4. 31336439 10.1016/j.dsx.2019.01.016

[29] Bechmann LE Emanuelsson F Nordestgaard BG Benn M. SGLT2-inhibition increases total, LDL, and HDL cholesterol and lowers triglycerides: meta-analyses of 60 randomized trials, overall and by dose, ethnicity, and drug type. Atherosclerosis. 2024;394:117236. 37582673 10.1016/j.atherosclerosis.2023.117236

[30] Kamijo Y Ishii H Yamamoto T. Potential impact on lipoprotein subfractions in type 2 diabetes. Clin Med Insights Endocrinol Diabetes. 2019;12:1179551419866811. 31452606 10.1177/1179551419866811 PMC6696845

[31] Liu S Ke J Feng X. The effect of canagliflozin on high-density lipoprotein cholesterol and angiopoietin-like protein 3 in type 2 diabetes mellitus. J Diabetes Res. 2024;2024:2431441. 38577301 10.1155/2024/2431441 PMC10994702

[32] Jojima T Sakurai S Wakamatsu S. Empagliflozin increases plasma levels of campesterol, a marker of cholesterol absorption, in patients with type 2 diabetes: association with a slight increase in high-density lipoprotein cholesterol. Int J Cardiol. 2021;331:243–8. 33556413 10.1016/j.ijcard.2021.01.063

[33] Lee MH Neeland IJ de Albuquerque Rocha N Hughes C Malloy CR Jin ES. A randomized clinical trial evaluating the effect of empagliflozin on triglycerides in obese adults: role of visceral fat. Metabol Open. 2022;13:100161. 35024596 10.1016/j.metop.2021.100161 PMC8728102

[34] Miklankova D Markova I Hüttl M Malinska H. Empagliflozin alters lipid metabolism in the myocardium and liver in a prediabetes model with severe dyslipidemia. Front Pharmacol. 2024;15:1393946. 39027339 10.3389/fphar.2024.1393946 PMC11254829

[35] Ge M Molina J Kim JJ. Empagliflozin reduces podocyte lipotoxicity in experimental Alport syndrome. Elife. 2023;12:e83353. 37129368 10.7554/eLife.83353 PMC10185338

[36] Klekowski J Chabowski M Krzystek-Korpacka M Fleszar M. The utility of lipidomic analysis in colorectal cancer diagnosis and prognosis—a systematic review of recent literature. Int J Mol Sci. 2024;25:7722. 39062964 10.3390/ijms25147722 PMC11277303

[37] Haycock PC Borges MC Burrows K. The association between genetically elevated polyunsaturated fatty acids and risk of cancer. EBioMedicine. 2023;91:104510. 37086649 10.1016/j.ebiom.2023.104510 PMC10148095

[38] Bhatt K Orlando T Meuwis MA Louis E Stefanuto PH Focant JF. Comprehensive insight into colorectal cancer metabolites and lipids for human serum: a proof-of-concept study. Int J Mol Sci. 2023;24:9614. 37298566 10.3390/ijms24119614 PMC10253775

[39] Pickens CA Pereira MFA Fenton JI. Long-chain ω-6 plasma phospholipid polyunsaturated fatty acids and association with colon adenomas in adult men: a cross-sectional study. Eur J Cancer Prev. 2017;26:497–505. 27768609 10.1097/CEJ.0000000000000312

[40] Dąbek A Wojtala M Pirola L Balcerczyk A. Modulation of cellular biochemistry, epigenetics and metabolomics by ketone bodies. implications of the ketogenic diet in the physiology of the organism and pathological states. Nutrients. 2020;12:788. 32192146 10.3390/nu12030788 PMC7146425

[41] Li C Wang F Cui L Li S Zhao J Liao L. Association between abnormal lipid metabolism and tumor. Front Endocrinol (Lausanne). 2023;14:1134154. 37305043 10.3389/fendo.2023.1134154 PMC10248433

